# The Therapeutic Potential and Clinical Significance of Exosomes as Carriers of Drug Delivery System

**DOI:** 10.3390/pharmaceutics15010021

**Published:** 2022-12-21

**Authors:** Tianwei Li, Xiaoqing Li, Guiping Han, Ming Liang, Zongrui Yang, Congyi Zhang, Shizhuan Huang, Sheng Tai, Shan Yu

**Affiliations:** 1 Department of Hepatic Surgery, Second Affiliated Hospital of Harbin Medical University, Harbin 150000, China; 2 Department of Pathology, Second Affiliated Hospital of Harbin Medical University, Harbin 150000, China; 3 Department of Infectious Diseases, Second Affiliated Hospital of Harbin Medical University, Harbin 150000, China

**Keywords:** exosome, drug delivery system, exosome carriers, tumor, CNS disease

## Abstract

Drug delivery system (DDS) realizes the drug delivery process through the drug carrier. As an important part of DDS, the selection of the drug carrier material is extremely critical, which requires the carrier material to possess excellent biocompatibility and targeting and not affect the pharmacological action of the drug. As one of the endogenous extracellular vesicles, exosomes are 30–100 nm in diameter, which are considered a new generation of a natural nanoscale delivery system. Exosomes secreted by different types of cells carry signaling molecules (such as proteins and nucleic acid) playing an important role in cell behaviors. Owing to their ability to specialize in intercellular communication, exosomes provide a distinctive method to deliver therapeutic drugs to target cells. In this concept, exosomes as the natural liposomes carry endogenous biomolecules, have excellent biocompatibility, and could be loaded with cargo both in vivo and in vitro. In addition, modifications by genetic and/or chemical engineering to part of the exosome surface or complement the desired natural effect may enhance the targeting with drug loading capability. Notably, exosomes weakly react with serum proteins prolonging cargo half-life. Overall, exosomes as natural carriers integrate the superiority of synthetic nanocarriers and cellular communication while precluding their limitations, which provides novel and reliable methods for drug delivery and treatment. Our review focuses on the therapeutic potentials and clinical values of exosomes as a carrier of drug delivery system in multiple diseases, including cancer, nervous, immune, and skeletal system diseases.

## 1. Introduction

Exosomes are biphospholipid bodies formed from membranes and emitted by cells [1]. In 1983, exosomes were discovered in supernatant from cultured sheep erythrocytes and were considered as secreted garbage [2]. Following in-depth investigations revealed that exosomes could be released by tumor cells, immune cells, mesenchymal stem cells, and endothelial progenitor cells and are widely detectable in blood, cerebrospinal fluid, saliva, ascites, and even urine [3]. Now, it is generally accepted that exosomes are 30–100 nm in diameter, rich in lipids (cholesterol, sphingomyelin, and ceramide), and fulfilled with active molecules including DNA, noncoding RNA, mRNA or protein (Table 1) [4]. Those unique tissue- or cell-specific proteins and genetic materials help to identify their cellular origin and the status of parental cells. Moreover, exosomes widely participate in biological activities such as cell communication, migration, angiogenesis, immunomodulation, and proliferation. During the exploiting their delivery function, exosomes from human donor cells are found to be capable of transferring chemical drugs or endogenous substances (proteins, mRNAs, miRNAs, lipids, etc.) to receiver cells for therapy. Even plant-derived exosomes have been characterized and shown to have structures similar to mammalian exosomes to mediate plant–animal intercellular communication [5]. Therefore, exosomes as naturally derived biological carriers have been refined into the concept of drug delivery. Due to the possible immune responses and lack of targeting, current studies on heterologous exosomes lack their clinical application, we mainly reviewed the role of homologous exosomes as drug delivery carriers. As an emerging drug delivery carrier, exosomes loaded with chemical or genetic drugs play a promising therapeutic role in diseases.

## 2. Formation and Understanding of Exosomes

### 2.1. Mechanism in the Secretion of Exosomes

**Figure 1:**
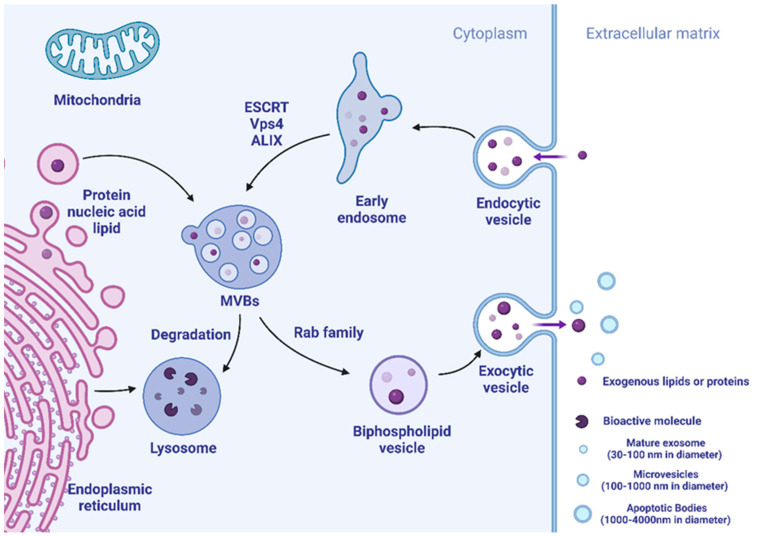
The illustration of exosome exchange. Bioactive components can be absorbed into the cell through endocytosis. Vesicle formation is considered to generate early endosomes, which are in turn shaped as MVBs. Part of MVBs could be degraded in lysosomes or through autophagosomes, while other parts could be returned to the plasma membrane and be secreted to the extracellular matrix, forming exosomes.

Exosomes are mainly formed by endocytosis of cell membranes, followed by converting to multivesicular bodies (MVBs) with dynamic subcellular architecture. Parts of MVBs could be fused to lysosomes, and their contents are degraded or recycled. The remaining MVBs may merge with the membrane followed by secretion to the extracellular matrix to form new extracellular vesicles including exosomes. The biogenesis mechanism of exosomes is controlled by related proteins, including annexin and tetraspanins (CD63). The endosomal sorting complex required for transport (ESCRT) is accepted as molecular media by which exosomes have specific cargo sorting functions [12]. Studies have shown that ESCRT-0, ESCRT-I, and ESCRT-II may be regulating cargo sorting, and ESCRT-III is involved in membrane endocytosis and exocytosis. Beginning with ESCRT-0, early endosomes for cargo-specific are initiated, recruited, and assembled to eventually form MBV [13,14]. In addition, Vps4 and ALIX also participate in cargo sorting and exosome formation [15,16]. Exosome formation steps are summarized in Figure 1.

In addition, exosomes are regulated by cytokines growth factors, proteins, distinct cell types, and other physicochemical conditions at multiple stages in the production process and widely exist in cells and body fluids in vivo, making them well-tolerated and -regulated [17].

### 2.2. Using Exosome Carriers in Drug Delivery System for Therapeutics

The drug carrier is the key component of a successful DDS. With the progress of science and technology, artificial materials such as synthetic liposomes and polylactic acid have been exploited as carriers in the last few years. Small volume, large surface area, and high-dose drug loading are the common characteristics of these materials [18,19]. However, these materials have the following disadvantages: low transduction efficiency, lack of target specificity, rapid degradation, and toxicity. The ideal drug carrier should have the characteristics of sustained, controlled, and targeted release; be compatible with the host immune system, specifically absorbed by target cells; and maintain a sufficient circulating half-life with the loaded drug. This requires the carrier material to have appropriate biocompatibility and biodegradability without affecting drug efficacy [20]. At present, exosomes have the above conditions perfectly. As a natural liposome in the human body, exosomes are considered to have the capability of high-efficiency loading and co-transmission of therapeutic drugs [21]. Thus far, the cargo biological component is fulfilled in exosomes through incubation, sonication, freeze-thaw cycling, extrusion, and electroporation once extracted from the donor cells [22]. In vivo, an effective way to modify exosomes is by up-regulating a gene in the parental cells or culturing cell lines with a chemical drug. The gene or drug will then be gently encapsulated in vesicles according to the process of exosome biogenesis [23]. Another promising feature is that simple surface modifications by bioengineering allow exosome delivery cargo more targeted. Although exosome carriers are natural, surface modifications can be performed easily with strategies that include genetic or/and chemical engineering modification. 

In genetic-related engineering approach, nucleic acids molecules ligands are fused to exosome surface proteins genetically and overexpressed subsequently in donor cells. The study by Zou et al. demonstrated that the constructs containing the platelet-derived growth factor receptor (PDGFR) transmembrane domain fused to single-chain variable fragments of human immunodeficiency virus (HIV)-1 Env-specific antibodies have been used to target exosomes to HIV-1-infected cells [24]. Similarly, one promising example is that Mentkowski et al. generated a highly efficient exosome delivery system that could target cardiomyocytes by modifying exosomes from cardiomyocytes (CDCs). The researchers fused cardiomyocyte targeting peptide (CMP) to the N-terminus of Lamp2b (a mouse transmembrane protein) through exosomes. After intramyocardial injection, they found that CMP-targeted CDC-derived exosomes could help in increasing cardiomyocyte uptake, decreasing apoptosis, and promoting cardiac retention. Making the groundwork supported exosomes for specific-targeting cells by drugs and gene therapy [25]. While the chemical methods rely on the bioconjugation of protein or chemical ligands to surface proteins. For example, Kim et al. used paclitaxel (PTX)-loaded exosomes with incorporated aminoethylanisamide-polyethylene glycol (AA-PEG) vector moiety to aim at binding with the sigma receptor, increasing the anticancer efficacy of PTX in lung cancer cells. Their result showed that AA-modified vesicles had a better loading capacity and therapeutic efficiency in vivo [26]. Similarly, Gao et al. used a phage-identified peptide (CP05) covalently bound to the exosome surface protein CD63. Following systemic administration of CP05-loaded exosomes, researchers found that targeting CP05-loaded exosomes increased the delivery of splice-correcting oligomers to muscle, thereby restoring muscular dystrophy [27]. It is clear that the above two major modification strategies make exosomes extremely advantageous when delivering drugs as carriers.

### 2.3. Advantages of Exosome Carriers for Drug Delivery

**Figure 2:**
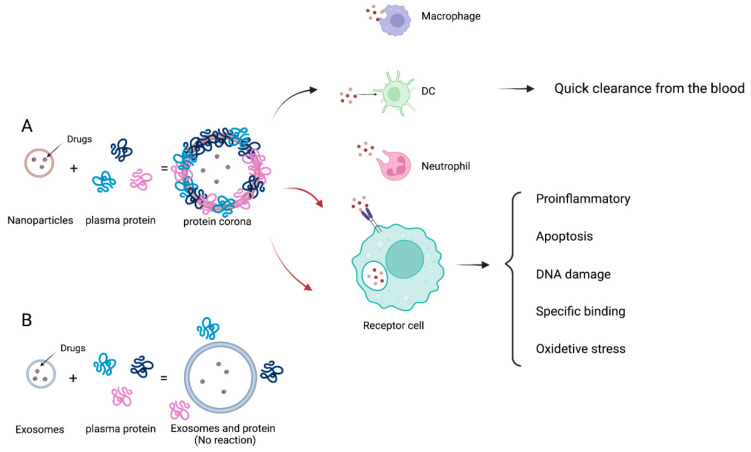
The schematic representation of the biological reaction of the protein crown in blood circulation. (**A**) Various plasma proteins adsorb onto NPs immediately after the synthetic NPs enter the blood, resulting in rapid clearance and cytotoxicity; (**B**) exosomes weakly bind with the serum proteins, thereby enhancing the circulation time and decreasing the cytotoxicity.

Compared with artificially synthesized carriers, the advantages of exosomes as carriers of DDS are generally known as follows: (1) A plentiful number of proteins and genetic molecules as a component in exosomes indicate that the great majority of biological substances can be loaded without unexpected interactions with the carrier. Endogenous exosomes have limited therapeutic efficacy, but exogenous exosomes can load dissimilar cargoes, including reconstituted proteins or therapeutic nucleic acids. They have superior compatibility that is critical to their function as carriers [28], which also shows a tremendous inherent capacity for receiver cells [29]. (2) Exosomes could be detected in almost any body fluids (blood, cerebrospinal fluid, saliva, etc.), indicating their transport cargo could go through membrane and be protected from degradations [30]. Furthermore, those nature-made vesicles are better tolerated in vivo. (3) The powerful targeted delivery capability and homing ability of exosomes as DDS carriers for therapeutic aims: First, exosomes can modulate targeting capabilities through loading specific binding principles of receptors and ligands, for example, dendritic cells (DC)-derived exosomes could transfer major histocompatibility complexes to antigen-presenting cell for regulating immunity [31]. Second, the targeted homing potential and cellular uptake capacity of exosomes are enhanced with the presence of vesicle surface proteins. Studies have shown that cancer-cell-derived exosomes tend to merge with their parental cells preferentially. This phenomenon created novel targeted therapies based on exosomes to deliver antitumor drugs [32]. Furthermore, integrins on exosomes determine the organotropism effect of exosomes into specific tissues. (4) Unlike synthetic liposomes, exosomes can be loaded with cargo in vivo by transfection and in vitro by electroporation and lipofection [33]. (5) Exosomes show faint nonspecific interactions with serum proteins. It was reported that serum proteins bind on the surface of synthetic nanoparticle (NP) carriers immediately after entering the bloodstream, forming so-called protein coronas [34,35]. The protein crowns can affect the properties of synthetic NP such as cell targeting, cell interactions, toxicity, etc. Furthermore, the protein corona causes an immune response and is quickly cleared from the blood (Figure 2) [36,37]. Notably, the binding of serum proteins with exosomes is weak, mainly based on exosome’s endogenous nature. Therefore, exosomes have good biocompatibility as carriers, which can prolong the circulation half-time with therapeutic cargo [38].

With the above advantages, exosomes are capable to refrain from phagocytosis and cross biological barriers with therapeutic substances (proteins, mRNAs, lipids, chemical drugs) to sites that are difficult to arrive at by artificial carriers. For instance, exosomes can cross the blood–brain barrier (BBB) and transport specific drugs to the neural cell [39], significantly retaining side effects including toxicity. With the promising and highly efficient carry potentials in DDS, exosomes are showing good biocompatibility, non-immunogenicity, and biodegradability and intensively investigated in the therapeutic role for different diseases.

## 3. Tumors

Exosomes rich in genetic material, proteins, and lipids may carry effective communication information for donor cells and target cells, which may build up the tumor microenvironment (TME) [40]. Tumor-derived exosomes (TDEs) were also found to have strong associations with TME and cancer initiation. Therefore, TDEs could be devised as natural drug carriers with higher cancer cell targeting and therapeutic efficacy, low toxicity, and permeability [41].

### 3.1. Delivering Endogenous Biomolecules 

Endogenous biomolecules contain abundant regulatory substances such as hormones, antibodies, cytokines, etc., which can also be incorporated into TDEs for therapeutic effects. Exosomes may provide a more rational way for therapeutic protein delivery to exert antitumor effects as we highlighted above [42]. In pancreatic cancer, it was shown that exosomes loaded with the survival protein-T34A protein induced apoptosis and enhanced the gemcitabine-killing effect [43]. Nie et al. synthesized exosome nano-protein bioconjugates for tumor therapy by conjugating azide-modified exosomes from M1 macrophages to dibenzocyclooctyne-modified antibodies to CD47 and SIRPα via a pH-sensitive linker. Their result showed that the nano-bioconjugates could specifically bind with cancer by recognition of CD47. Meanwhile, native M1 exosomes efficiently reprogram macrophages from pro-tumor M2 to anti-tumor M1 [44]. Furthermore, strong immunogenic proteins embedded in exosomes may provoke immune activation to inhibit cancer proliferation with the potential for developing cancer vaccines. Notably, DC-derived exosomes gain MHC-I that combines with tumor-derived peptides, and the complex could activate NK or T cells to suppress tumors [45,46]. In addition to proteins, nucleic acids (mRNA, miRNA, siRNA, piRNA, etc.) have also been reported as a potential method for cancer treatment [47,48]. In Yang et al. research, mouse embryonic fibroblast-derived exosomes were able to deliver PTEN (phosphatase and homologous tensin) mRNA to inhibit mouse glioma growth [49]. Furthermore, exosome transport solves the problem of easy degradation of miRNAs in vivo, implying a new tumor therapy [50]. Brien et al. reported that mesenchymal stem cells (MSC)-derived exosomes containing miR-379 were shown to have a therapeutic effect on breast cancer, with significantly reduced tumor growth rates in T47D breast cell line expressing miR-379 [51]. In addition, exosomes from MSCs had a higher amount of miR-1455p were displaying a strong effect on the inhibition of pancreatic cancer cells in vitro and in vivo [52]. Similarly, siRNA delivery can also be used for cancer treatment via post-transcriptional gene silencing for targeting cells. Shtam et al. showed the therapeutic potential of exosome-mediated siRNA delivery to HeLa cells (cervical cancer cell line) by targeting RAD51. These results demonstrate the cancer therapeutic capacity of using exosome carriers to deliver nucleic acids for RNAi-based gene therapy [53]. Furthermore, genetic tools can also be embedded into TDEs such as plasmids. Kim et al. used exosomes secreted from SKOV3 as a vector to express the CRISPR/Cas9 plasmid. The CRISPR/Cas9-loaded exosomes significantly down-regulated PARP-1 expression, thus causing ovarian cancer cell death [54].

### 3.2. Delivering Chemical Drugs

Antitumor drugs such as vincristine, curcumin, PTX, camptothecin, etc., most of which are monomer active ingredients extracted from Chinese medicine, generally have disadvantages including poor stability, low solubility, high hydrophobicity, and short half-life, etc. Therefore, their low bioavailability limited the clinical application. The efficacy of these monomeric components can be enhanced by the administration of exosome carriers [55]. For example, Saari et al. showed that tumor cell-derived exosomes could be considered a useful vehicle for paclitaxel from the parental cell, carrying the drug into recipient cells through the endocytosis pathway and increasing their cytotoxicity [56]. Similarly, Garofalo et al. showed lung tumor cell-derived exosomes can effectively carry the oncolytic virus and chemotherapeutic drugs (PTX), thus enhancing the antitumor effects in nude mice [57]. Tang et al. showed another example that exosomes pre-loaded with cisplatin could significantly decrease liver or kidney toxicities and suppress tumors in ovarian cancer mice xenograft model [58]. In general, exosome-loaded drugs display better efficacy than chemicals used alone.

### 3.3. Delivering Engineered Drugs

In recent years, researchers have increasingly turned to transport conjugated or modified drugs through TDEs to promote therapeutic efficacy. This procedure generally involves the reconstitution of multiple drugs through encapsulation [59]. For example, the researchers used mouse macrophage exosomes to hybridize with synthetic liposomes to obtain engineered exosomes, water-soluble doxorubicin loaded in the engineered exosomes under acidic conditions had a significantly enhanced toxicity to cancer cells. It strongly suggests that the engineered exosomes will be a promising platform for tumor-targeted drug delivery [60]. Yong et al. have innovated a biocompatible exogenous tumor-cell-exocytosed exosome-biomimetic porous silicon nanoparticles (PSiNPs) as a drug carrier for anti-tumor. After being pre-loaded with Doxorubicin, the exosome sheath enhanced tumor accumulation and vascular extravasation. In addition, this system exhibits remarkable cell absorption and cytotoxicity in tumor cells [61]. Similarly, Wu et al. synthesized sequential nano catalysts GOD-ESIONs@EVs (GE@EVs) by mixing HCC-derived exosomes as surface nanocarriers with nano-scale iron oxide particles solution (ESIONS)-arginine-glycine-aspartic acid (RGD). The RGD parts are accounting for membrane fusion to enhance cellular endocytosis and the sequential nano catalysts underwent a more effective treatment in the HCC tumor zone during a short time. In addition, Jia et al. used materials such as superparamagnetic iron oxide nanoparticles (SPION) and curcumin to coat a novel glioma-targeting exosome. Their findings demonstrated that the engineered exosome strongly improved the treatment of glioma while reducing side effects [62]. The above studies provide validations for the application of exosome carriers-loaded engineered drugs for high targeting and efficient anticancer activity.

**Figure 3:**
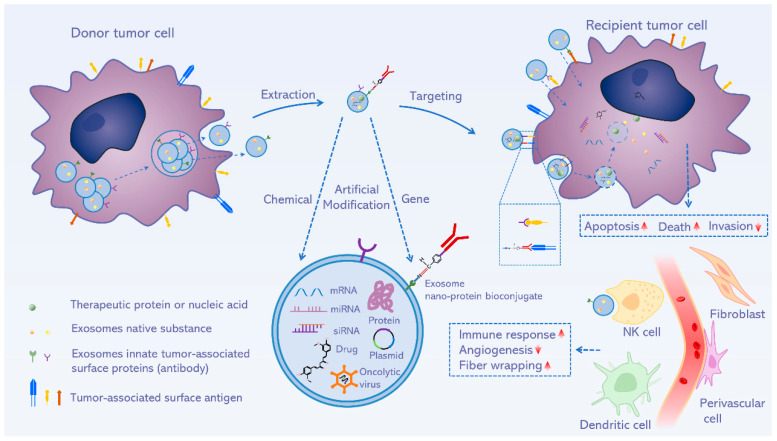
Schematic illustration of the tumor-derived exosomes with modification strategies for enhancing targeting. A chemical or gene modification of extracted exosomes from tumor cells may further promote the anti-tumor functions from indicated concepts.

In summary, TDEs contain tumor-associated antigens that can preferably identify tumors and targeted transmit therapeutic substances. Therefore, exosome carriers possess superior biocompatibility, causing drugs more easily uptake by the cells and avoiding drug resistance (Figure 3). Additionally, exosome carriers could hold the drug stability and half-life.

## 4. Neurological Diseases

In the central nervous system (CNS), exosomes have a role in mediating intercellular communication, maintaining homeostasis, and neuroprotective (Table 2). Furthermore, exosome carriers can deliver therapeutic substances across the blood–brain barrier (BBB). Therefore, it opens up a promising possibility to deliver therapeutic substances to treat central neurological diseases, such as neurodegenerative diseases, strokes, etc. [63].

For example, traumatic brain injury (TBI) can cause severe neuronal damage, and studies have shown that exosomes from human-induced pluripotent stem cells (hiPSCs) and MSCs may heal neuronal damage [68]. Moon et al. studied the biological distribution, therapeutic effect, and action mode of MSC-derived exosomes in a rat stroke model. The results illustrated strong evidence for MSC-derived exosomes could successfully stimulate neurogenesis and angiogenesis in that model [69].

The engineered DCs-derived exosomes with Lamp2b had been generated and combined into a neuron-specific RVG peptide. After extraction of the exosome, an exogenous siRNA was loaded. Notably, exosomes specifically delivered loaded cargo to neurons in the brain, downregulating the siRNA target gene and impacted on Alzheimer’s disease (AD) [70]. Others reported that exosome carriers conjugated to the surface of c (RGDyK) peptide or curcumin can target the lesion area of ischemic encephalopathy. In a mouse model, the modified exosomes provided a safe and effective delivery vehicle for ischemic stroke therapy [71]. Wang et al. studied engineered exosomes that silenced Bcl-2 and Bax with a promising result that suggested the exosomes had a therapeutic effect on apoptosis after TBI [72].

In addition, emerging engineering techniques are applied to modify the exosome to further promote its capacity to carry cargo. Ferrantelli et al. identified a negative regulatory factor mutant (Nefmut) of the HIV-encoded. The researchers loaded Nefmut into exosomes and fused a single-chain fragment variable antibody at its C-terminus to form engineered exosomes, which proposed a novel targeted strategy for endogenous engineering in the treatment of neurodegenerative diseases based on exosomes released by the cellular composition of the CNS [73]. Qu et al. established a biocompatible kit for the exosome-based delivery system across the BBB. In a mouse Parkinson’s disease (PD) model, exosomes with pre-loaded dopamine exhibited better performance than free dopamine after intravenous injection [74]. Haney et al. developed engineered exosomes loaded with catalase (ExoCAT), and after intranasal administration, a major proportion of exosomes could be found in the brains of PD mice. In addition, ExoCAT showed effective neuroprotection in vivo models of PD [75]. With the development of engineered and advanced loading cargo technology, exosomes as DDS carriers will certainly have greater therapeutic potential and value for CNS diseases in the future.

## 5. Autoimmune Diseases

Previous studies have proved that natural or surface-modified exosomes can be used as a treatment for autoimmune diseases [76]. As conventional treatments for autoimmune diseases suffer from low bioavailability, rapid clearance, limited targeting ability, and poor therapeutic outcomes due to the unfavorable pharmacokinetic behavior of the drugs, exosomes carriers can efficiently load biologics or other inflammation inhibitors and deliver them to the target protected from enzyme-caused degradation, which provides tantalizing prospects in supporting the fight against autoimmune diseases. It was reported that miR-146a/miR-155-transduced MSC-derived exosome strongly increased Treg cell subpopulation and anti-inflammatory cytokines in collagen-induced arthritis (CIA) mice. Ultimately, this modulation may facilitate the restoration of T-cell responses in rheumatoid arthritis (RA); therefore, the use of MSC-derived exosomes to load miRNAs that can alter immune responses could serve as therapeutic targets for inflammatory diseases [77]. Riazifar et al. showed that exosome-stimulated MSCs produced by γ-interferon have been shown to have significant effects in the treatment of autoimmune encephalomyelitis (EAE) [78].

Studies have shown that glucocorticoid-loaded exosomes show therapeutic efficacy in proteolipoprotein-induced experimental autoimmune encephalomyelitis. Animals treated with glucocorticoid-loaded exosomes recovered from acute illness more rapidly than clinically used multiple sclerosis (MS) drugs, such as betafalon and copasone [79]. To suppress the side effects of methotrexate, researchers encapsulated the chemical drug in exosomes from MSCs, and it showed better retention and a 10-fold inhibition of inflammation compared to methotrexate alone [80]. Similarly, Kadry et al. found the glutathione-loaded exosomes were more able to shorten the glutathione synthesis process, increase glutathione serum levels, and meanwhile reduce rheumatoid factors. The prognostic marker malondialdehyde and C-reactive protein levels were lower than the free glutathione group [81].

Furthermore, exosomes can be modified with biological or synthetic ligands on the unique surface to enhance the targeting specific possibility to deliver cargo in different autoimmune diseases. For example, Yan et al. established biomimetic exosomes and modified them with compounds such as folic acid (FA), polyethylene glycol (PEG), and cholesterol to obtain an actively targeted drug delivery vehicle loaded with dexamethasone sodium phosphate. In vivo biodistribution experiments showed that these engineered exosomes better protected bone and cartilage in CIA mice and reduced joint inflammation without obvious liver toxicity. This approach can avoid the side effects of using corticosteroids alone [82]. Similarly, a murine microglial cell line secreted engineered exosomes loaded with IL-4 was designed by Casella. After applying the above exosomes, the experimental autoimmune encephalomyelitis score decreased sharply, significantly reducing tissue damage. The engineered exosomes can also deliver a variety of functional molecules to treat inflammatory diseases [83]. Exosomes show promising prospects for the treatment of autoimmune diseases.

## 6. Other Systemic Diseases

### 6.1. Skeletal Diseases

Traditional drug therapy for diseases of the skeletal system is mainly based on inhibiting osteoclast formation (estrogen, etc.) or inhibiting osteoclast activity (bisphosphonates, etc.). However, the prolonged use of high doses of drugs that can cause secondary adverse effects, including affecting normal osteocytes [84]. Clearly, bone drug delivery applications are not yet perfect in targeting and eliminating side effects. Due to the advent of exosome carriers, great progress in the management of drug delivery to the bone has been made [85]. Zha et al. extracted ATDC5 (chondrogenic progenitor cell line)-derived exosomes to load the vascular endothelial growth factor (VEGF) gene, which can effectively restore segmental bone defects by increasing osteogenesis and angiogenesis [86]. The result shows inspiring evidence for curing segmental bone defects. Luo et al. showed that bone marrow stromal cell (ST)-derived exosomes (STExos) could significantly enhance the osteoblast differentiation of bone mesenchymal stem cells (BMSCs) in vitro by simple surface chemical modifications. Intravenous administration of STExos could not improve osteoporotic phenotypes in mouse models. The researchers found significantly higher efficiency after conjugating the STExo surface with BMSC-specific aptamers, confirming the novel modification could help in osteoporosis and fractures [87]. Moreover, the treatment of the engineered bone scaffolds with exosomes as carriers not only provides direct bracing for bone defects but also provides a suitable status for bone regeneration. In addition, Zhang et al. found MSC-exosome/β-TCP composite scaffolds had better healing functions on rat calvarial defects [88]. Another study showed that targeting Kartogenin (KGN) to synovial fluid-derived mesenchymal stem cells (SF-MSCs) by engineered exosomes resulted in uniform dispersion of KGN in the cytoplasmic matrix, increased concentration in the target, and significantly promoted chondrogenesis of SF-MSCs. In a rat model, KGN loaded by engineered exosomes also showed better therapeutic effects than KGN alone [89]. In the future, with the further elucidation of the biological information contained in exosomes, as a natural carrier, exosomes will be able to deliver drugs more precisely and efficiently and exhibit a promising role in the treatment of bone defects, delayed healing, and other skeletal system diseases.

### 6.2. Cardiovascular Diseases

Cardiovascular exosomes also have a significant clinical role and therapeutic value. All cell subtypes in the cardiovascular system, including endothelial cells (ECs), cardiomyocytes, and fibroblasts, release exosomes to participate in intercellular communication and regulate cardiac function. Therefore, the superior biocompatibility of exosomes allows them potentially useful as drug carriers in cardiovascular diseases [90]. Liu et al. investigated the role of exosomes loaded with miR-19a, miR-210, and other miRNAs against oxidative stress (OS) in coronary heart disease, and the results suggest that the ability of exosomes to target loading endogenous antioxidants may make them a more effective treatment for OS than stem cell therapy [91]. In addition, exosomes with angiogenesis-related miRNAs were able to ameliorate cardiac function. For example, injecting miR-19a-3p-loaded exosomes into mouse myocardium revealed that exosomes resulted in improved angiogenesis, decreased myocardial fibrosis, and increased left ventricular ejection fraction [92]. Similarly, Chen et al. showed that bone marrow-secreted MSC exosomes carrying miRNA-125b could prevent myocardial ischemia-reperfusion injury by targeting SIRT7 [93]. A c(RGDyK) peptide-conjugated, cholesterol-modified miR-210-engineered exosomes showed up-regulated expression of integrinβ3, VEGF, and CD34 and demonstrated that miR-210 for cerebral hematogenous delivery provides angiogenic agents for alleviating ischemic diseases [94]. Wu et al. reported that molecularly engineered M2 macrophage-derived exosomes may contribute to treating atherosclerosis by suppressing inflammation via chemokine receptors and inflammation inhibitory cytokines secreted by M2 macrophages. Meanwhile, the encapsulated hexyl 5-aminolevulinate hydrochloride can diminish inflammation effectively by intrinsic biosynthesis and metabolism of heme [95]. Notably, Zhang et al. constructed novel biomimetic nanobubbles (Mel@NVs) composed of exosome-loaded melatonin (Mel) from adipose-derived stem cells (ADSCs). Further reports showed the Mel@NVs could promote the formation of microvessels and alleviate cardiac fibrosis, thereby further restoring mitochondrial dysfunction for myocardial repair. Mel@NVs proved to be a potentially promising therapy for myocardial infarction [96]. As a DDS carrier, exosome performs ideally when delivering therapeutic substances in cardiovascular diseases [97].

### 6.3. Urinary System Diseases

Traditionally, the signaling of cytokines and inflammatory mediators has been recognized as an important player in the pathogenesis of kidney diseases. It has been reported that exosomes can participate in renal tissue injury and regeneration by mediating inter-nephron communication [98]. Notably, exosomes are used as carriers to load therapeutic substances such as proteins and RNAs with extremely high stability, which provides an attractive method as novel therapeutic carriers for kidney disease treatment. For example, nuclear factor (NF)-ĸB signaling significantly participated in acute kidney injury induced by ischemia-reperfusion. Researchers used a new and optogenetically engineered exosome technology called EXPLOR to deliver exosomes with effective NF-ĸB inhibitors into a kidney ischemia-reperfusion mice model. Compared with the control group, the results confirmed it could improve renal injury significantly by regulating different biological components [99]. In another study, M1-Exo-GEM (pre-loading M1 macrophage-derived exosomes(M1-Exo) with Gemcitabine (GEM) by ultrasound technology) was built up for killing mouse bladder cancer MB49 cells. Compared with M1-Exo and GEM, M1-Exo-GEM had significantly up-regulated the expression of inflammatory cytokines and stronger cytotoxic effect on cancer cells [100].

### 6.4. Cutaneous Disease

Previous studies have revealed that exosomes could be used as a new therapeutic option for skin repair and regeneration treatment [101]. For example, exosomes derived from human umbilical vein endothelial cells (HUVECs) can promote collagen maturation and angiogenesis and regulate the keratinocytes and fibroblast growth, thereby accelerating skin wound healing [102]. Similarly, Zhang et al. found that exosomes from adipose tissue-derived stem cells can accelerate collagen synthesis and redeposition through regulating PI3K/Akt pathway. This function helps in full-thickness skin wounds, ultimately reducing healing time and scarring in mouse models [103]. In addition, exosomes can be further designed to deliver therapeutic cargos to treat skin diseases. For example, recent evidence suggests that the transport of ncRNA (including miRNA and lncRNA) target cells through the role of exosomes in the pathophysiology of psoriatic arthritis [104]. Furthermore, Shiekh et al. have shown that an antioxidant wound dressing, consisting of antioxidant polyurethane combined with ADSCs extracted exosomes, could shorten the diabetic wound healing time [105]. Similarly, Wang et al. loaded epidermal stem cell (ESC)-derived exosomes with VH298 (VH-EV), and VH-EV was shown to promote the function of HUVECs in vitro through HIF-1α signaling. This special exosome displayed great therapeutic effects on skin regeneration [106]. In addition, as carriers, exosomes provide an emerging delivery method for therapeutic miRNAs that neither penetrate the skin nor easily cross cell membranes. Xia et al. found that exosome-based miR-125b may accelerate myofibroblast differentiation and transfer to young fibroblast by targeting SIRT7. The results show a promising method for counteracting skin aging [107].

### 6.5. Metabolic Disease

The role of exosomes as agents for drug delivery in metabolic diseases is worth investigating [108]. However, major studies are focused on the complications of metabolic diseases, such as the diabetic skin complications discussed above [109,110]. At the same time, engineered exosomes also have broad potential in the treatment of obesity. Guo et al. designed an engineered smart exosome platform called SmartExo@Bmp7. This biological system induced local white adipose tissue browning through transporting Bmp7 mRNA to reduce obesity for C57BL/6 mice. As proof, this evidence opens a novel anti-obesity therapy [111].

## 7. The Conclusion and Future Breakthrough Directions of Exosome Carriers

In recent years, exosome has received special attention for its therapeutic values. A growing number of studies have proven that exosomes play a key role in the occurrence and development of diseases. Thus far, the clinical application of exosomes is mainly used for body fluid biopsy and clinical diagnosis of diseases such as cancer, nervous system diseases. and immune diseases [112]. However, the achievements are still limited by their novelty. In 2016, the world’s first cancer diagnostic product based on exosome miRNAs body fluid biopsy, ExoDx Lung (ALK), was launched, which could detect EML4-ALK mutations in patients with non-small cell lung cancer in real time [113]. Similarly, Zhang et al. showed that exosome microRNAs (such as miR-193a-3p, miR-210-3p, and miR-5100) from hypoxic BMSCs were selectively taken up by lung cancer cells and could activate STAT3 signaling to induce epithelial–mesenchymal transition (EMT) to promote the invasion of lung cancer cells, which has been identified as a new biomarker for lung cancer progression [114].

Additionally, as an ideal natural cargo carrier, exosomes also have great therapeutic value in almost all types of diseases. Exosomes secreted by cells in different tissues carry different biological components, conferring unique biological roles, which enable exosomes to deliver substances such as chemical drugs, nucleic acids, and proteins, etc., to perform the therapeutic role with distinguishable advantages (Table 3). Those advantages include a higher safety profile that escapes from immune detection systems; a better-tolerated and longer-circulating half-life, which prolongs the cargo from degradation; a more easy targeting ability, by which the exosomes’ surface could be artificially modified to increase biological effects as carriers and targeted transport; and easy penetration of cell membranes and biological barriers, including the BBB, due to their nanometer size and specific surface molecules.

However, most of the research on exosome carriers is still in the vitro stage. The criteria for the manufacture, purification, storage, duration of stability, loading efficiency, and dosage of exosomes also require further investigations to optimize and break through [125,126]. Moreover, existing results enhanced the interaction of exosomes with their targets by modifying exosome surface proteins, but they did not counteract the natural homing ability of exosomes, nor did they prevent off-target effects. There is currently a lack of standardized testing for drugs delivered by exosomes and also few studies investigating the ultimate fate of exosomes. Therefore, more research on the uptake mechanism of exosomes is necessary to ensure that they can deliver cargo directly to the targeted cytoplasm and exert therapeutic effects [127]. In addition, while previous studies have yielded important findings for exosome therapy, most of them only involve in vitro models, so it is unclear whether these results reflect in vivo processes. Furthermore, most of these studies simply analyzed a certain number of miRNAs or proteins. There is a lack of understanding of the exosomes with abundant biological information from distinct cell types. Although several databases of exosome contents exist, diversities between studies and techniques require extraction and purification standardization to elucidate contents and ultimately increase the diversity of cargo molecules [128]. The physical characteristics of the exosome (including size, shape, surface charge, and density) are necessary to determine its role and function. Currently, there is still a great limitation in accurately detecting these characteristics and purity [129]. Therefore, existing findings require careful analysis, and further research in this area is required to accurately measure these physicochemical properties and isolate exosomes from complex biological fluids. Commonly used isolation techniques, such as nanoparticle tracking analysis (NTA), dynamic light scattering (DLS), resistive pulse sensing (RPS), etc., cannot optimally separate exosomes and non-exosomes pollutants and still require extensive experiments studies to improve the purity of exosomes [130]. At the same time, challenges remain in reliably tracking exosomes in vivo, and this needs to be verified by extensive experiments using high-purity exosomes in vitro. The preparation of an in vitro exosome delivery cargo model may be a new method to solve this problem. In addition, the scalability or reproducibility of exosome production is also one of the challenges. Although MSCs can generate a great amount of exosomes, it requires a long time to obtain an effective therapeutic amount [131]. Similar studies are needed in the future to expand other cells or to speed up the collection of specific types of cells. Finally, the translations of exosomes into clinics are confined within existing regulatory frameworks. Therefore, these limitations are required to be fixed for the final application to the clinic.

Briefly, as we mentioned above, exosomes are involved in the occurrence and progression of various systemic diseases, which determine their intrinsic properties of high biocompatibility and low immunogenicity. As a new generation of drug delivery carriers, exosomes could deliver therapeutic drugs to target cells in a targeted manner, playing a therapeutic role in curing diseases. However, methods to regulate cargo packaging and vesicle release in vivo are still limited. Additionally, some technical, functional, and safety features of exosome-based pharmaceutical formulations have not yet been resolved. More safety validations are still required before exosome carriers could be widely performed clinically. Future research on exosomes may focus on scale-up of production and quality control and rigorous pharmacokinetic and reducing toxicology studies before eventually clinical trials. The achievement of these goals will lay the groundwork for the subsequent industrial development of exosomes as novel therapeutic interventions. In short, no difficulties could retard the emerging role of exosomes as a successful player in novel biological drug delivery systems for future clinical applications.

## Figures and Tables

**Table 1:** Summary of the exosome compositions.

	Compositions		Reference
Protein	Cell surface protein	MHCI, MHCII, CD63, CD81, CD9, Lamp-2b, lactadherin	[6]
	Cytoplasmic proteins	Ubiquitin, ALIX, ESCRT, HSC70, HSP70, HSP60, HSP90, etc.	[7]
	Cytoskeletal protein	Actin, tubulin, Keratin 8, 10, 18 and 19, α-Actinin-4	[8]
	Enzymes	Lysosomal, AAA ATPase, kinases, fatty acid synthase	[9]
Nucleic acid	mRNA, miRNA, ncRNA, siRNA		[10]
Lipids	Ceramide, sphingomyelin, phosphatidylserine, phosphatidylcholine, steroid lipids, etc.		[11]

**Table 2:** Mechanisms of exosome neuroprotection.

Exosome Sources	The Main Molecules	Molecules or Pathways Acting in the Process	The Main Mechanism	Reference
Mesenchymal stem cells (MSC)	N/A	N/A	Increased angiogenesis after ischemia	[64]
Adipose-derived MSCs	miR-25-3p	Autophagy	Reduce neuronal autophagy	[65]
The astrocyte origin	circSHOC2	miR-7670-3p/SIRT1	Inhibition of neuronal cell apoptosis	[66]
The astrocyte origin	microRNA-34c	TLR7, NF-κB/MAPK	Reduce ischemia/reperfusion (I/R) injury	[67]

**Table 3:** Exosomes derived from sources.

Exosome Sources	Function	Reference
Mesenchymal stem cells	Promote tolerant immune responses, suppress inflammatory responses	[115]
Cancer cell	Involved in tumor microenvironment and cancer development	[116]
Macrophage	Intercellular communication	[117]
Dendritic cells	Immune stimulation	[118]
Mast cell	Participate in immune regulation	[119]
Other cells (such as human amniotic epithelial cells, endothelial progenitor cells, etc.)	Reflect the pathophysiological state of their cell origin and can be used to diagnose and predict disease	[120,121]
Body fluid (saliva, ascites, etc.)	Biomarker and therapeutic agent for diseases	[122]
Heterogeneity (coconut, ginger, pea, bovine or caprine milk, etc.)	Mediate plant–animal intercellular communication	[123,124]

## Data Availability

Not applicable.

## References

[1] Boyiadzis M., Whiteside T.L. (2015). Information transfer by exosomes: A new frontier in hematologic malignancies. Blood Rev..

[2] Pan B.-T., Johnstone R.M. (1983). Fate of the transferrin receptor during maturation of sheep reticulocytes in vitro: Selective externalization of the receptor. Cell.

[3] Lässer C., Alikhani V.S., Ekström K., Eldh M., Paredes P.T., Bossios A., Sjöstrand M., Gabrielsson S., Lötvall J., Valadi H. (2011). Human saliva, plasma and breast milk exosomes contain RNA: Uptake by macrophages. J. Transl. Med..

[4] Kowal J., Tkach M., Théry C. (2014). Biogenesis and secretion of exosomes. Curr. Opin. Cell Biol..

[5] Zhang M., Viennois E., Xu C., Merlin D. (2016). Plant derived edible nanoparticles as a new therapeutic approach against diseases. Tissue Barriers.

[6] Pathan M., Fonseka P., Chitti S.V., Kang T., Sanwlani R., Van Deun J., Hendrix A., Mathivanan S. (2019). Vesiclepedia 2019: A compendium of RNA, proteins, lipids and metabolites in extracellular vesicles. Nucleic Acids Res..

[7] Liang Y., Duan L., Lu J., Xia J. (2021). Engineering exosomes for targeted drug delivery. Theranostics.

[8] Kowal J., Arras G., Colombo M., Jouve M., Morath J.P., Primdal-Bengtson B., Dingli F., Loew D., Tkach M., Théry C. (2016). Proteomic comparison defines novel markers to characterize heterogeneous populations of extracellular vesicle subtypes. Proc. Natl. Acad. Sci. USA.

[9] Jeppesen D.K., Fenix A.M., Franklin J.L., Higginbotham J.N., Zhang Q., Zimmerman L.J., Liebler D.C., Ping J., Liu Q., Evans R. (2019). Reassessment of Exosome Composition. Cell.

[10] Ragusa M., Barbagallo C., Cirnigliaro M., Battaglia R., Brex D., Caponnetto A., Barbagallo D., Di Pietro C., Purrello M. (2017). Asymmetric RNA Distribution among Cells and Their Secreted Exosomes: Biomedical Meaning and Considerations on Diagnostic Applications. Front. Mol. Biosci..

[11] Xu X., Xu L., Zhang P., Ouyang K., Xiao Y., Xiong J., Wang D., Liang Y., Duan L. (2020). Effects of ATP9A on Extracellular Vesicle Release and Exosomal Lipid Composition. Oxidative Med. Cell. Longev..

[12] Li T., Li J., Wang H., Zhao J., Yan M., He H., Yu S. (2022). Exosomes: Potential Biomarkers and Functions in Head and Neck Squamous Cell Carcinoma. Front. Mol. Biosci..

[13] Horváth P., Müller-Reichert T. (2020). A Structural View on ESCRT-Mediated Abscission. Front. Cell Dev. Biol..

[14] Zhen Y., Spangenberg H., Munson M.J., Brech A., Schink K.O., Tan K.-W., Sørensen V., Wenzel E.M., Radulovic M., Engedal N. (2020). ESCRT-mediated phagophore sealing during mitophagy. Autophagy.

[15] Jackson C.E., Scruggs B.S., Schaffer J.E., Hanson P.I. (2017). Effects of Inhibiting VPS4 Support a General Role for ESCRTs in Extracellular Vesicle Biogenesis. Biophys. J..

[16] Larios J., Mercier V., Roux A., Gruenberg J. (2020). ALIX- and ESCRT-III–dependent sorting of tetraspanins to exosomes. J. Cell Biol..

[17] Meldolesi J. (2018). Exosomes and Ectosomes in Intercellular Communication. Curr. Biol..

[18] Higa L.H., Jerez H.E., de Farias M.A., Portugal R.V., Romero E.L., Morilla M.J. (2017). Ultra-small solid archaeolipid nanoparticles for active targeting to macrophages of the inflamed mucosa. Nanomedicine.

[19] Martínez-Carmona M., Lozano D., Colilla M., Vallet-Regí M. (2018). Lectin-conjugated pH-responsive mesoporous silica nanoparticles for targeted bone cancer treatment. Acta Biomater..

[20] Allen T.M., Cullis P.R. (2004). Drug Delivery Systems: Entering the Mainstream. Science.

[21] Yang M., Wu S.Y. (2018). The Advances and Challenges in Utilizing Exosomes for Delivering Cancer Therapeutics. Front. Pharmacol..

[22] Mehryab F., Rabbani S., Shahhosseini S., Shekari F., Fatahi Y., Baharvand H., Haeri A. (2020). Exosomes as a next-generation drug delivery system: An update on drug loading approaches, characterization, and clinical application challenges. Acta Biomater..

[23] Familtseva A., Jeremic N., Tyagi S.C. (2019). Exosomes: Cell-created drug delivery systems. Mol. Cell. Biochem..

[24] Zou X., Yuan M., Zhang T., Wei H., Xu S., Jiang N., Zheng N., Wu Z. (2019). Extracellular vesicles expressing a single-chain variable fragment of an HIV-1 specific antibody selectively target Env+ tissues. Theranostics.

[25] Mentkowski K., Lang J.K. (2019). Exosomes Engineered to Express a Cardiomyocyte Binding Peptide Demonstrate Improved Cardiac Retention in Vivo. Sci. Rep..

[26] Kim M.S., Haney M.J., Zhao Y., Yuan D., Deygen I., Klyachko N.L., Kabanov A.V., Batrakova E.V. (2018). Engineering macrophage-derived exosomes for targeted paclitaxel delivery to pulmonary metastases: In vitro and in vivo evaluations. Nanomedicine.

[27] Gao X., Ran N., Dong X., Zuo B., Yang R., Zhou Q., Moulton H.M., Seow Y., Yin H. (2018). Anchor peptide captures, targets, and loads exosomes of diverse origins for diagnostics and therapy. Sci. Transl. Med..

[28] Wei H., Chen Q., Lin L., Sha C., Li T., Liu Y., Yin X., Xu Y., Chen L., Gao W. (2021). Regulation of exosome production and cargo sorting. Int. J. Biol. Sci..

[29] Anand S., Samuel M., Kumar S., Mathivanan S. (2019). Ticket to a bubble ride: Cargo sorting into exosomes and extracellular vesicles. Biochim. Biophys. Acta Proteins Proteom..

[30] Lai R.C., Yeo R.W.Y., Tan K.H., Lim S.K. (2013). Exosomes for drug delivery—A novel application for the mesenchymal stem cell. Biotechnol. Adv..

[31] Liu Q., Rojas-Canales D.M., DiVito S.J., Shufesky W.J., Stolz D.B., Erdos G., Sullivan M.L., Gibson G.A., Watkins S.C., Larregina A.T. (2016). Donor dendritic cell–derived exosomes promote allograft-targeting immune response. J. Clin. Investig..

[32] Qiao L., Hu S., Huang K., Su T., Li Z., Vandergriff A., Cores J., Dinh P.-U., Allen T., Shen D. (2020). Tumor cell-derived exosomes home to their cells of origin and can be used as Trojan horses to deliver cancer drugs. Theranostics.

[33] Xi X.-M., Xia S.-J., Lu R. (2021). Drug loading techniques for exosome-based drug delivery systems. J. Pharm. Sci..

[34] Peng Q., Mu H. (2016). The potential of protein–nanomaterial interaction for advanced drug delivery. J. Control. Release.

[35] Müller J., Prozeller D., Ghazaryan A., Kokkinopoulou M., Mailänder V., Morsbach S., Landfester K. (2018). Beyond the protein corona—Lipids matter for biological response of nanocarriers. Acta Biomater..

[36] Xiao W., Wang Y., Zhang H., Liu Y., Xie R., He X., Zhou Y., Liang L., Gao H. (2021). The protein corona hampers the transcytosis of transferrin-modified nanoparticles through blood-brain barrier and attenuates their targeting ability to brain tumor. Biomaterials.

[37] Sutaria D.S., Badawi M., Phelps M.A., Schmittgen T.D. (2017). Achieving the Promise of Therapeutic Extracellular Vesicles: The Devil is in Details of Therapeutic Loading. Pharm. Res..

[38] Fernandes M., Lopes I., Teixeira J., Botelho C., Gomes A.C. (2020). Exosome-like Nanoparticles: A New Type of Nanocarrier. Curr. Med. Chem..

[39] Zhang Y., Chopp M., Meng Y., Katakowski M., Xin H., Mahmood A., Xiong Y. (2015). Effect of exosomes derived from multipluripotent mesenchymal stromal cells on functional recovery and neurovascular plasticity in rats after traumatic brain injury. J. Neurosurg..

[40] Li I., Nabet B.Y. (2019). Exosomes in the tumor microenvironment as mediators of cancer therapy resistance. Mol. Cancer.

[41] Tarasov V.V., Svistunov A.A., Chubarev V.N., Dostdar S.A., Sokolov A.V., Brzecka A., Sukocheva O., Neganova M.E., Klochkov S.G., Somasundaram S.G. (2021). Extracellular vesicles in cancer nanomedicine. Semin. Cancer Biol..

[42] Yim N., Ryu S.W., Choi K., Lee K.R., Lee S., Choi H., Kim J., Shaker M.R., Sun W., Park J.H. (2016). Exosome engineering for efficient intracellular delivery of soluble proteins using optically reversible protein-protein interaction module. Nat. Commun..

[43] Aspe J.R., Diaz Osterman C.J., Jutzy J.M., Deshields S., Whang S., Wall N.R. (2014). Enhancement of Gemcitabine sensitivity in pancreatic adenocarcinoma by novel exosome-mediated delivery of the Survivin-T34A mutant. J. Extracell. Vesicles.

[44] Nie W., Wu G., Zhang J., Huang L., Ding J., Jiang A., Zhang Y., Liu Y., Li J., Pu K. (2019). Responsive Exosome Nano-bioconjugates for Synergistic Cancer Therapy. Angew. Chem. Int. Ed..

[45] Hartman Z.C., Wei J., Glass O.K., Guo H., Lei G., Yang X.-Y., Osada T., Hobeika A., Delcayre A., Le Pecq J.-B. (2011). Increasing vaccine potency through exosome antigen targeting. Vaccine.

[46] Romagnoli G.G., Zelante B.B., Toniolo P.A., Migliori I.K., Barbuto J.A.M. (2015). Dendritic Cell-Derived Exosomes may be a Tool for Cancer Immunotherapy by Converting Tumor Cells into Immunogenic Targets.. Front. Immunol..

[47] Geis-Asteggiante L., Belew A.T., Clements V.K., Edwards N.J., Ostrand-Rosenberg S., El-Sayed N.M., Fenselau C. (2018). Differential Content of Proteins, mRNAs, and miRNAs Suggests that MDSC and Their Exosomes May Mediate Distinct Immune Suppressive Functions. J. Proteome Res..

[48] Dvorská D., Braný D., Ňachajová M., Halašová E., Danková Z. (2021). Breast Cancer and the Other Non-Coding RNAs. Int. J. Mol. Sci..

[49] Yang Z., Shi J., Xie J., Wang Y., Sun J., Liu T., Zhao Y., Zhao X., Wang X., Ma Y. (2020). Large-scale generation of functional mRNA-encapsulating exosomes via cellular nanoporation. Nat. Biomed. Eng..

[50] Ell B., Mercatali L., Ibrahim T., Campbell N., Schwarzenbach H., Pantel K., Amadori D., Kang Y. (2013). Tumor-Induced Osteoclast miRNA Changes as Regulators and Biomarkers of Osteolytic Bone Metastasis. Cancer Cell.

[51] O’Brien K.P., Khan S., Gilligan K., Zafar H., Lalor P., Glynn C., O’Flatharta C., Ingoldsby H., Dockery P., De Bhulbh A. (2018). Employing mesenchymal stem cells to support tumor-targeted delivery of extracellular vesicle (EV)-encapsulated microRNA-379. Oncogene.

[52] Ding Y., Cao F., Sun H., Wang Y., Liu S., Wu Y., Cui Q., Mei W., Li F. (2019). Exosomes derived from human umbilical cord mesenchymal stromal cells deliver exogenous miR-145-5p to inhibit pancreatic ductal adenocarcinoma progression. Cancer Lett..

[53] Shtam T.A., Kovalev R.A., Varfolomeeva E.Y., Makarov E.M., Kil Y.V., Filatov M.V. (2013). Exosomes are natural carriers of exogenous siRNA to human cells in vitro. Cell Commun. Signal..

[54] Kim S.M., Yang Y., Oh S.J., Hong Y., Seo M., Jang M. (2017). Cancer-derived exosomes as a delivery platform of CRISPR/Cas9 confer cancer cell tropism-dependent targeting. J. Control. Release.

[55] Farooqi A.A., Desai N.N., Qureshi M.Z., Librelotto D.R.N., Gasparri M.L., Bishayee A., Nabavi S.M., Curti V., Daglia M. (2018). Exosome biogenesis, bioactivities and functions as new delivery systems of natural compounds. Biotechnol. Adv..

[56] Saari H., Lázaro-Ibáñez E., Viitala T., Vuorimaa-Laukkanen E., Siljander P., Yliperttula M. (2015). Microvesicle- and exosome-mediated drug delivery enhances the cytotoxicity of Paclitaxel in autologous prostate cancer cells. J. Control. Release.

[57] Garofalo M., Saari H., Somersalo P., Crescenti D., Kuryk L., Aksela L., Capasso C., Madetoja M., Koskinen K., Oksanen T. (2018). Antitumor effect of oncolytic virus and paclitaxel encapsulated in extracellular vesicles for lung cancer treatment. J. Control. Release.

[58] Tang K., Zhang Y., Zhang H., Xu P., Liu J., Ma J., Lv M., Li D., Katirai F., Shen G.-X. (2012). Delivery of chemotherapeutic drugs in tumour cell-derived microparticles. Nat. Commun..

[59] Palazzolo S., Memeo L., Hadla M., Duzagac F., Steffan A., Perin T., Canzonieri V., Tuccinardi T., Caligiuri I., Rizzolio F. (2020). Cancer Extracellular Vesicles: Next-Generation Diagnostic and Drug Delivery Nanotools. Cancers.

[60] Rayamajhi S., Nguyen T.D.T., Marasini R., Aryal S. (2019). Macrophage-derived exosome-mimetic hybrid vesicles for tumor targeted drug delivery. Acta Biomater..

[61] Yong T., Zhang X., Bie N., Zhang H., Zhang X., Li F., Hakeem A., Hu J., Gan L., Santos H.A. (2019). Tumor exosome-based nanoparticles are efficient drug carriers for chemotherapy. Nat. Commun..

[62] Jia G., Han Y., An Y., Ding Y., He C., Wang X., Tang Q. (2018). NRP-1 targeted and cargo-loaded exosomes facilitate simultaneous imaging and therapy of glioma in vitro and in vivo. Biomaterials.

[63] Ghosh S., Ghosh S. (2022). Exosome: The “Off-the-Shelf” Cellular Nanocomponent as a Potential Pathogenic Agent, a Disease Biomarker, and Neurotherapeutics. Front. Pharmacol..

[64] Doeppner T.R., Herz J., Görgens A., Schlechter J., Ludwig A.-K., Radtke S., de Miroschedji K., Horn P.A., Giebel B., Hermann D.M. (2015). Extracellular Vesicles Improve Post-Stroke Neuroregeneration and Prevent Postischemic Immunosuppression. Stem Cells Transl. Med..

[65] Kuang Y., Zheng X., Zhang L., Ai X., Venkataramani V., Kilic E., Hermann D.M., Majid A., Bähr M., Doeppner T.R. (2020). Adipose-derived mesenchymal stem cells reduce autophagy in stroke mice by extracellular vesicle transfer of miR-25. J. Extracell. Vesicles.

[66] Chen W., Wang H., Zhu Z., Feng J., Chen L. (2020). Exosome-Shuttled circSHOC2 from IPASs Regulates Neuronal Autophagy and Ameliorates Ischemic Brain Injury via the miR-7670-3p/SIRT1 Axis. Mol. Ther. Nucleic Acids.

[67] Wu W., Liu J., Yang C., Xu Z., Huang J., Lin J. (2020). Astrocyte-derived exosome-transported microRNA-34c is neuroprotective against cerebral ischemia/reperfusion injury via TLR7 and the NF-κB/MAPK pathways. Brain Res. Bull..

[68] Ghosh S., Garg S., Ghosh S. (2020). Cell-Derived Exosome Therapy: A Novel Approach to Treat Post-traumatic Brain Injury Mediated Neural Injury. ACS Chem. Neurosci..

[69] Moon G.J., Sung J.H., Kim D.H., Kim E.H., Cho Y.H., Son J.P., Cha J.M., Bang O.Y. (2019). Application of Mesenchymal Stem Cell-Derived Extracellular Vesicles for Stroke: Biodistribution and MicroRNA Study. Transl. Stroke Res..

[70] Alvarez-Erviti L., Seow Y., Yin H., Betts C., Lakhal S., Wood M.J. (2011). Delivery of siRNA to the mouse brain by systemic injection of targeted exosomes. Nat. Biotechnol..

[71] Tian T., Zhang H.-X., He C.-P., Fan S., Zhu Y.-L., Qi C., Huang N.-P., Xiao Z.-D., Lu Z.-H., Tannous B.A. (2018). Surface functionalized exosomes as targeted drug delivery vehicles for cerebral ischemia therapy. Biomaterials.

[72] Wei H., Chen J., Wang S., Fu F., Zhu X., Wu C., Liu Z., Zhong G., Lin J. (2019). A Nanodrug Consisting of Doxorubicin And Exosome Derived From Mesenchymal Stem Cells For Osteosarcoma Treatment In Vitro. Int. J. Nanomed..

[73] Ferrantelli F., Chiozzini C., Leone P., Manfredi F., Federico M. (2020). Engineered Extracellular Vesicles/Exosomes as a New Tool against Neurodegenerative Diseases. Pharmaceutics.

[74] Qu M., Lin Q., Huang L., Fu Y., Wang L., He S., Fu Y., Yang S., Zhang Z., Zhang L. (2018). Dopamine-loaded blood exosomes targeted to brain for better treatment of Parkinson’s disease. J. Control. Release.

[75] Haney M.J., Klyachko N.L., Zhao Y., Gupta R., Plotnikova E.G., He Z., Patel T., Piroyan A., Sokolsky M., Kabanov A.V. (2015). Exosomes as drug delivery vehicles for Parkinson’s disease therapy. J. Control. Release.

[76] Zhou X., Xie F., Wang L., Zhang L., Zhang S., Fang M., Zhou F. (2020). The function and clinical application of extracellular vesicles in innate immune regulation. Cell. Mol. Immunol..

[77] Tavasolian F., Hosseini A.Z., Soudi S., Naderi M. (2020). miRNA-146a Improves Immunomodulatory Effects of MSC-derived Exosomes in Rheumatoid Arthritis. Curr. Gene Ther..

[78] Riazifar M., Mohammadi M.R., Pone E.J., Yeri A., Lässer C., Segaliny A.I., McIntyre L.L., Shelke G.V., Hutchins E., Hamamoto A. (2019). Stem Cell-Derived Exosomes as Nanotherapeutics for Autoimmune and Neurodegenerative Disorders. ACS Nano.

[79] Avnir Y., Turjeman K., Tulchinsky D., Sigal A., Kizelsztein P., Tzemach D., Gabizon A., Barenholz Y. (2011). Fabrication Principles and Their Contribution to the Superior In Vivo Therapeutic Efficacy of Nano-Liposomes Remote Loaded with Glucocorticoids. PLoS ONE.

[80] Neupane Y.R., Mahtab A., Siddiqui L., Singh A., Gautam N., Rabbani S.A., Goel H., Talegaonkar S. (2020). Biocompatible Nanovesicular Drug Delivery Systems with Targeting Potential for Autoimmune Diseases. Curr. Pharm. Des..

[81] Kadry M.O. (2019). Liposomal glutathione as a promising candidate for immunological rheumatoid arthritis therapy. Heliyon.

[82] Yan F., Zhong Z., Wang Y., Feng Y., Mei Z., Li H., Chen X., Cai L., Li C. (2020). Exosome-based biomimetic nanoparticles targeted to inflamed joints for enhanced treatment of rheumatoid arthritis. J. Nanobiotechnol..

[83] Casella G., Colombo F., Finardi A., Descamps H., Ill-Raga G., Spinelli A.E., Podini P., Bastoni M., Martino G., Muzio L. (2018). Extracellular Vesicles Containing IL-4 Modulate Neuroinflammation in a Mouse Model of Multiple Sclerosis. Mol. Ther..

[84] Kennel K.A., Drake M.T. (2009). Adverse effects of bisphosphonates: Implications for osteoporosis management. Mayo. Clin. Proc..

[85] Yang X., Chen S., Liu X., Yu M., Liu X. (2019). Drug Delivery Based on Nanotechnology for Target Bone Disease. Curr. Drug Deliv..

[86] Zha Y., Li Y., Lin T., Chen J., Zhang S., Wang J. (2021). Progenitor cell-derived exosomes endowed with VEGF plasmids enhance osteogenic induction and vascular remodeling in large segmental bone defects. Theranostics.

[87] Luo Z.-W., Li F.-X., Liu Y.-W., Rao S.-S., Yin H., Huang J., Chen C.-Y., Hu Y., Zhang Y., Tan Y.-J. (2019). Aptamer-functionalized exosomes from bone marrow stromal cells target bone to promote bone regeneration. Nanoscale.

[88] Zhang J., Liu X., Li H., Chen C., Hu B., Niu X., Li Q., Zhao B., Xie Z., Wang Y. (2016). Exosomes/tricalcium phosphate combination scaffolds can enhance bone regeneration by activating the PI3K/Akt signaling pathway. Stem. Cell Res. Ther..

[89] Xu X., Liang Y., Li X., Ouyang K., Wang M., Cao T., Li W., Liu J., Xiong J., Li B. (2021). Exosome-mediated delivery of kartogenin for chondrogenesis of synovial fluid-derived mesenchymal stem cells and cartilage regeneration. Biomaterials.

[90] Liu Q., Piao H., Wang Y., Zheng D., Wang W. (2021). Circulating exosomes in cardiovascular disease: Novel carriers of biological information. Biomed. Pharmacother..

[91] Liu Y., Wang M., Liang Y., Wang C., Naruse K., Takahashi K. (2021). Treatment of Oxidative Stress with Exosomes in Myocardial Ischemia. Int. J. Mol. Sci..

[92] Gollmann-Tepeköylü C., Pölzl L., Graber M., Hirsch J., Nägele F., Lobenwein D., Hess M.W., Blumer M.J., Kirchmair E., Zipperle J. (2010). miR-19a-3p containing exosomes improve function of ischaemic myocardium upon shock wave therapy. Cardiovasc. Res..

[93] Chen Q., Liu Y., Ding X., Li Q., Qiu F., Wang M., Shen Z., Zheng H., Fu G. (2020). Bone marrow mesenchymal stem cell-secreted exosomes carrying microRNA-125b protect against myocardial ischemia reperfusion injury via targeting SIRT7. Mol. Cell. Biochem..

[94] Zhang H., Wu J., Wu J., Fan Q., Zhou J., Wu J., Liu S., Zang J., Ye J., Xiao M. (2019). Exosome-mediated targeted delivery of miR-210 for angiogenic therapy after cerebral ischemia in mice. J. Nanobiotechnol..

[95] Wu G., Zhang J., Zhao Q., Zhuang W., Ding J., Zhang C., Gao H., Pang D.-W., Pu K., Xie H.-Y. (2020). Molecularly Engineered Macrophage-Derived Exosomes with Inflammation Tropism and Intrinsic Heme Biosynthesis for Atherosclerosis Treatment. Angew. Chem. Int. Ed..

[96] Zhang Y., Yang N., Huang X., Zhu Y., Gao S., Liu Z., Cao F., Wang Y. (2022). Melatonin Engineered Adipose-Derived Biomimetic Nanovesicles Regulate Mitochondrial Functions and Promote Myocardial Repair in Myocardial Infarction. Front. Cardiovasc. Med..

[97] Lu M., Xing H., Xun Z., Yang T., Ding P., Cai C., Wang D., Zhao X. (2018). Exosome-based small RNA delivery: Progress and prospects. Asian J. Pharm. Sci..

[98] Bruno S., Porta S., Bussolati B. (2016). Extracellular vesicles in renal tissue damage and regeneration. Eur. J. Pharmacol..

[99] Kim S., A Lee S., Yoon H., Kim M.Y., Yoo J.-K., Ahn S.-H., Park C.H., Park J., Nam B.Y., Park J.T. (2021). Exosome-based delivery of super-repressor IκBα ameliorates kidney ischemia-reperfusion injury. Kidney Int..

[100] Tang Z., Tang C., Sun C., Ying X., Shen R. (2022). M1 macrophage-derived exosomes synergistically enhance the anti- bladder cancer effect of gemcitabine. Aging.

[101] Wang W., Wu C., Jin H. (2019). Exosomes in chronic inflammatory skin diseases and skin tumors. Exp. Dermatol..

[102] Zhao D., Yu Z., Li Y., Wang Y., Li Q., Han D. (2020). GelMA combined with sustained release of HUVECs derived exosomes for promoting cutaneous wound healing and facilitating skin regeneration. Histochem. J..

[103] Zhang W., Bai X., Zhao B., Li Y., Zhang Y., Li Z., Wang X., Luo L., Han F., Zhang J. (2018). Cell-free therapy based on adipose tissue stem cell-derived exosomes promotes wound healing via the PI3K/Akt signaling pathway. Exp. Cell Res..

[104] Yue T., Ji M., Qu H., Guo M., Bai F., Zhang Z., Wang W., Gong X., Zhang Z. (2019). Comprehensive analyses of long non-coding RNA expression profiles by RNA sequencing and exploration of their potency as biomarkers in psoriatic arthritis patients. BMC Immunol..

[105] Shiekh P.A., Singh A., Kumar A. (2020). Exosome laden oxygen releasing antioxidant and antibacterial cryogel wound dressing OxOBand alleviate diabetic and infectious wound healing. Biomaterials.

[106] Wang Y., Cao Z., Wei Q., Ma K., Hu W., Huang Q., Su J., Li H., Zhang C., Fu X. (2022). VH298-loaded extracellular vesicles released from gelatin methacryloyl hydrogel facilitate diabetic wound healing by HIF-1α-mediated enhancement of angiogenesis. Acta Biomater..

[107] Xia W., Li M., Jiang X., Huang X., Gu S., Ye J., Zhu L., Hou M., Zan T. (2022). Young fibroblast-derived exosomal microRNA-125b transfers beneficial effects on aged cutaneous wound healing. J. Nanobiotechnol..

[108] Warshauer J.T., Bluestone J.A., Anderson M.S. (2020). New Frontiers in the Treatment of Type 1 Diabetes. Cell Metab..

[109] Xu Y.-X., Pu S.-D., Li X., Yu Z.-W., Zhang Y.-T., Tong X.-W., Shan Y.-Y., Gao X.-Y. (2022). Exosomal ncRNAs: Novel therapeutic target and biomarker for diabetic complications. Pharmacol. Res..

[110] He C., Zheng S., Luo Y., Wang B. (2018). Exosome Theranostics: Biology and Translational Medicine. Theranostics.

[111] Guo Y., Wan Z., Zhao P., Wei M., Liu Y., Bu T., Sun W., Li Z., Yuan L. (2021). Ultrasound triggered topical delivery of Bmp7 mRNA for white fat browning induction via engineered smart exosomes. J. Nanobiotechnol..

[112] Zhou B., Xu K., Zheng X., Chen T., Wang J., Song Y., Shao Y., Zheng S. (2020). Application of exosomes as liquid biopsy in clinical diagnosis. Signal Transduct. Target. Ther..

[113] Zhang Y., Bi J., Huang J., Tang Y., Du S., Li P. (2020). Exosome: A Review of Its Classification, Isolation Techniques, Storage, Diagnostic and Targeted Therapy Applications. Int. J. Nanomed..

[114] Zhang X., Sai B., Wang F., Wang L., Wang Y., Zheng L., Li G., Tang J., Xiang J. (2019). Hypoxic BMSC-derived exosomal miRNAs promote metastasis of lung cancer cells via STAT3-induced EMT. Mol. Cancer.

[115] Xunian Z., Kalluri R. (2020). Biology and therapeutic potential of mesenchymal stem cell-derived exosomes. Cancer Sci..

[116] Kok V.C., Yu C.-C. (2020). Cancer-Derived Exosomes: Their Role in Cancer Biology and Biomarker Development. Int. J. Nanomed..

[117] Zheng Y., He R., Wang P., Shi Y., Zhao L., Liang J. (2019). Exosomes from LPS-stimulated macrophages induce neuroprotection and functional improvement after ischemic stroke by modulating microglial polarization. Biomater. Sci..

[118] Wahlund C.J., Güclüler G., Hiltbrunner S., Veerman R.E., Näslund T.I., Gabrielsson S. (2017). Exosomes from antigen-pulsed dendritic cells induce stronger antigen-specific immune responses than microvesicles in vivo. Sci. Rep..

[119] Xie G., Yang H., Peng X., Lin L., Wang J., Lin K., Cui Z., Li J., Xiao H., Liang Y. (2018). Mast cell exosomes can suppress allergic reactions by binding to IgE. J. Allergy Clin. Immunol..

[120] Hadley E.E., Sheller-Miller S., Saade G., Salomon C., Mesiano S., Taylor R.N., Taylor B.D., Menon R. (2018). Amnion epithelial cell–derived exosomes induce inflammatory changes in uterine cells. Am. J. Obstet. Gynecol..

[121] Kong J., Wang F., Zhang J., Cui Y., Pan L., Zhang W., Wen J., Liu P. (2018). Exosomes of Endothelial Progenitor Cells Inhibit Neointima Formation After Carotid Artery Injury. J. Surg. Res..

[122] Lin J., Li J., Huang B., Liu J., Chen X., Chen X.-M., Xu Y.-M., Huang L.-F., Wang X.-Z. (2015). Exosomes: Novel Biomarkers for Clinical Diagnosis. Sci. World J..

[123] Xiao J., Feng S., Wang X., Long K., Luo Y., Wang Y., Ma J., Tang Q., Jin L., Li X. (2018). Identification of exosome-like nanoparticle-derived microRNAs from 11 edible fruits and vegetables. Peerj.

[124] Yun B., Kim Y., Park D.J., Oh S. (2021). Comparative analysis of dietary exosome-derived microRNAs from human, bovine and caprine colostrum and mature milk. J. Anim. Sci. Technol..

[125] Théry C., Witwer K.W., Aikawa E., Alcaraz M.J., Anderson J.D., Andriantsitohaina R., Antoniou A., Arab T., Archer F., Atkin-Smith G.K. (2018). Minimal information for studies of extracellular vesicles 2018 (MISEV2018): A position statement of the International Society for Extracellular Vesicles and update of the MISEV2014 guidelines. J. Extracell. Vesicles.

[126] Yáñez-Mó M., Siljander P.R.-M., Andreu Z., Bedina Zavec A., Borràs F.E., Buzas E.I., Buzas K., Casal E., Cappello F., Carvalho J. (2015). Biological properties of extracellular vesicles and their physiological functions. J. Extracell. Vesicles.

[127] Frolova L., Li I.T.S. (2022). Targeting Capabilities of Native and Bioengineered Extracellular Vesicles for Drug Delivery. Bioengineering.

[128] Abels E.R., Breakefield X.O. (2016). Introduction to Extracellular Vesicles: Biogenesis, RNA Cargo Selection, Content, Release, and Uptake. Cell. Mol. Neurobiol..

[129] Xie S., Zhang Q., Jiang L. (2022). Current Knowledge on Exosome Biogenesis, Cargo-Sorting Mechanism and Therapeutic Implications. Membranes.

[130] Tian Y., Gong M., Hu Y., Liu H., Zhang W., Zhang M., Hu X., Aubert D., Zhu S., Wu L. (2019). Quality and efficiency assessment of six extracellular vesicle isolation methods by nano-flow cytometry. J. Extracell. Vesicles.

[131] Bulut O., Gürsel İ. (2020). Mesenchymal stem cell derived extracellular vesicles: Promising immunomodulators against autoimmune, autoinflammatory disorders and SARS-CoV-2 infection. Turk. J. Biol..

